# “They Just Don’t Get Around to It”: Clinician Perspectives on Lung Cancer Screening and Racial Disparities Among Veterans

**DOI:** 10.1016/j.chpulm.2025.100165

**Published:** 2025-03-18

**Authors:** Neelima Navuluri, Govind Krishnan, Tiera Lanford, Abigail Shapiro, Angela B. Johnson, Isaretta L. Riley, Leah L. Zullig, Christopher E. Cox, Scott Shofer

**Affiliations:** a Division of Pulmonary, Allergy, and Critical Care, Department of Medicine, Duke University School of Medicine, Durham, NC; b Department of Medicine, Durham Veterans Affairs Medical Center, Durham, NC; c Duke Global Health Institute, Duke University, Durham, NC; d Center of Innovation to Accelerate Discovery and Practice Transformation (ADAPT), Durham Veteran Affairs Health Care System, Durham, NC; e Institute for Medical Research, Durham, NC; f Department of Population Health Sciences, Duke University, Durham, NC

**Keywords:** attitudes, early detection of cancer, health knowledge, lung cancer, practice, qualitative research, racial disparities, cancer screening, veterans

## Abstract

**Background:**

Lung cancer screening (LCS) rates are low across the United States, with substantial disparities in availability and uptake. This trend is also reflected in the Veterans Affairs Healthcare System. We aimed to understand clinician perspectives on factors leading to low uptake and racial disparities in LCS.

**Research Question:**

What are VA primary care providers' and LCS program staff's experiences with the LCS process and their perspectives on ways it could be improved to reduce disparities in LCS rates among Black veterans?

**Study Design and Methods:**

Semistructured interviews were conducted at a Southeastern US Veterans Affairs Healthcare System with primary care providers and LCS program staff. Interview questions and notetaking templates were developed using the Consolidated Framework for Implementation Research. Rapid qualitative analysis was used to assess perspectives on barriers, facilitators, and contextual factors leading to low uptake and racial disparities in LCS to help inform future interventions.

**Results:**

We interviewed 20 health care providers (17 primary care providers, 3 LCS program staff). Six emergent themes were derived from a combination of Consolidated Framework for Implementation Research domains and constructs. These included the following: (1) primary care providers’ complex experiences with the centralized LCS program, (2) LCS is 1 priority among many, (3) marked clinician variation in LCS shared decision-making and referral decisions, (4) racial biases and structural inequities, (5) limited clinician knowledge of patient-level screening facilitators, and (6) suggested program improvements. Themes underscored that improving the shared decision-making process and streamlining referral and scheduling are key areas for future interventions to improve LCS uptake.

**Interpretation:**

Providers reported multiple barriers contributing to LCS disparities and suggested targeting interventions to improve the shared decision-making process and the transition from primary care to LCS visit. Future studies should evaluate such interventions and their impact on LCS uptake and equity.

**Take-Home Points**

**Study Question:** What are Veteran Affairs primary care providers’ (PCPs) and lung cancer screening (LCS) program staff’s perspectives on barriers, facilitators, and contextual factors affecting disparities in LCS uptake?

**Results:** Interviews with PCPs and LCS staff identified the following 6 key themes: (1) PCPs’ complex experiences with the centralized LCS program, (2) LCS is 1 priority among many, (3) marked clinician variation in LCS shared decision-making and referral decisions, (4) racial biases and structural inequities, (5) limited clinician knowledge of patient-level screening facilitators, and (6) suggested program improvements.

**Interpretation:** This qualitative study identified several key barriers and facilitators to disparities in LCS uptake and highlighted potential patient-, provider-, and system-level interventions which could enhance equity in LCS.

Lung cancer screening (LCS) using annual low-dose CT scans has been shown to reduce lung cancer mortality by nearly 25% and is recommended by the National Comprehensive Cancer Network and US Preventive Services Task Force.^1^, ^2^, ^3^, ^4^ However, only 4.5% of eligible patients were screened across the United States in 2022.^5^ There are also significant disparities in LCS availability and uptake. Black patients are 2.8 times less likely to be screened compared with White patients, despite having a greater benefit from screening. Rural-dwelling patients are more likely to live > 48 kilometers from an accredited screening facility despite a greater smoking prevalence and higher lung cancer incidence.^6^

The Veterans Affairs Health Care System (VAHCS) is one of the largest integrated providers of cancer care in the United States and was an early implementer of LCS, providing it at no or minimal cost to veterans.^7^^,^^8^ Nonetheless, < 3% of eligible veterans have been screened. Disparities in LCS uptake within the VAHCS also exist. For example, a study demonstrated that 31% of Black veterans referred for screening received a CT scan compared with 41% of White veterans. National VAHCS data have shown wide variability in screening rates across states and rurality of residence.^9^^,^^10^

A systematic review of qualitative studies identified multiple patient-, provider-, and system-level barriers and facilitators of LCS across the United States.^11^ However, these studies did not focus on causes of and solutions for addressing racial disparities in LCS. We previously explored patient perspectives on barriers and facilitators to LCS among Black veterans.^12^ In this study, we aimed to understand VA primary care providers’ (PCPs) and LCS program staff’s perspectives on barriers, facilitators, and contextual factors to LCS uptake and identify ways the LCS process could be improved to reduce screening disparities among Black veterans.

## Study Design and Methods

### Study Design and Setting

We conducted a cross-sectional interview-based study in a Southeastern US VAHCS from February to June 2023. All study procedures were approved by the Durham VAHCS Institutional Review Board (Protocol No. 1622197). The study adhered to the Standards for Reporting Qualitative Research (e-Table 1).^13^

**Table 1:** Participant Characteristics (N = 20)

Characteristic	Value
Female sex	12 (60)
Self-reported race	
White/Caucasian	16 (80)
Asian	4 (20)
Black/African American	0 (0)
Self-reported ethnicity	
Not Hispanic or Latino	20 (100)
Clinician type	
Physician	13 (65)
Advanced practice provider	7 (35)
Participant type	
PCP	17 (85)
LCS program staff	3 (15)
PCP characteristics (n = 17)	
LCS referral rate	
≥ 1% of all referrals	9 (53)
< 1% of all referrals	8 (47)
Percent clinical time	80 (40-100)
Average No. of patients seen per week	30 (9-58)

Values are presented as No. (%) or median (quartile 1, quartile 3). LCS = lung cancer screening; PCP = primary care provider.

Durham VAHCS has a centralized LCS program which was established in 2013 as part of a national implementation project. It uses a consult model in which PCPs identify individuals who meet criteria for LCS and then place a consult for LCS. LCS program staff then review eligibility criteria again to ensure individual veterans do not have any contraindications to LCS (eg, another primary cancer, comorbidities which would make surgical resection infeasible). They then send out an informational mailer with a decision aid to eligible veterans who are instructed to call the program if interested. If a veteran agrees to LCS, program staff order a low-dose CT scan. These orders are restricted to LCS program staff, limiting the ability for PCPs or other providers to order these scans outside of the program.

Between 2013 and January 2025, the Durham VAHCS LCS program, which is in an urban area but serves an approximately 40% to 45% rural population, has received 9,058 referrals. Of those, 2,574 veterans have undergone LCS (LCS staff/patient screening ratio of 1:1,287), with a current total active screening population of 1,470 veterans. The central VA Lung Cancer Screening Program metrics dashboard indicates that there are 1,369 veterans eligible for screening who have not yet been referred.

### Participants and Recruitment

We interviewed attending PCPs and LCS program staff. A total of 100 PCPs referred veterans for screening at the site since 2012. This includes 80 attending PCPs and 60 resident PCPs. Given the relatively low number of LCS referrals among residents and the turnover every 3 years, we focused recruitment on attending PCPs who were identified via an LCS database at the local VAHCS. We used purposive sampling to ensure diversity in primary care sites and LCS referral rate. Referral rates were the number of referrals an individual PCP placed as a proportion of all LCS referrals within the local VAHCS, which ranged from 0.2% to 4.1% and had a median of 0.9%. Providers were thus stratified as ≥ 1% of all referrals (high) or < 1% of all referrals (low). LCS program staff were included as key informants given their integral involvement in the screening process and included the director of the LCS program and current screening coordinators. Prospective participants were sent emails informing them about the aims of the research and asking them to participate in a voluntary interview.

### Instrument Development and Data Collection

The Consolidated Framework for Implementation Research (CFIR) domains of individuals, innovation, outer setting, inner setting, and implementation process, and their individual constructs, informed the development of interview guides and analysis.^14^^,^^15^ Separate interview guides were developed for PCPs and LCS program staff; they asked about individual roles and characteristics, knowledge of the LCS process, shared decision-making, perceived barriers and facilitators to LCS, racial disparities in LCS, and recommendations for improving the LCS process (e-Table 1). Interview questions about racial disparities in LCS focused on knowledge of disparities and perceived barriers and facilitators to LCS for Black veterans. Semistructured interviews were conducted by qualitative analysts (T. L. and A. S.) using Microsoft Teams (Microsoft Inc) and were audio-recorded and auto-transcribed. Interviews lasted between 45 and 90 minutes.

### Data Analysis

Two clinicians, 1 female and 1 male (N. N. and G. K.), and 2 female analysts (T. L. and A. S.) analyzed interview data iteratively using the Hamilton and Finley approach^16^ to qualitative methods for implementation science. Interview transcripts and structured interviewer notes were reviewed, edited for clarity, and summarized into a matrix guided by interview questions and CFIR. Next, iterative memo development and matrix analyses were used to elucidate common and contrasting experiences and identify themes.^17^ Accuracy was confirmed by final review of transcripts and audio-recordings. Analytical disagreements were resolved through group discussion and consensus building.

### Patient and Public Involvement

Patients and the public were not involved in the design, reporting, or dissemination plans of this study.

## Results

We interviewed 17 PCPs and 3 LCS program staff; demographic characteristics are shown in Table 1. Generally, PCPs with high LCS referral rates spent ≥ 50% of their time in clinical practice and reported seeing at least 30 patients a week. PCPs with lower referral rates generally reported less clinical time.

**Figure 1:**
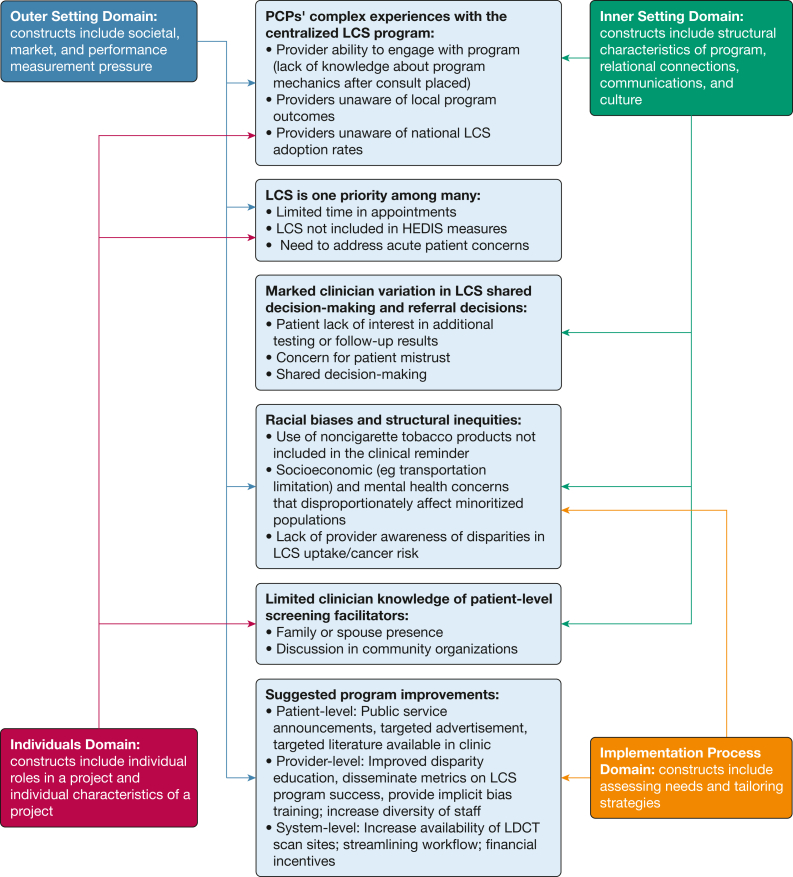
Mapping of Consolidated Framework for Implementation Research domains and selected constructs onto emergent themes. HEDIS = Healthcare Effectiveness Data and Information Set; LCS = lung cancer screening; LDCT = low-dose CT; PCP = primary care provider.

Six themes emerged across a combination of 4 CFIR domains (outer setting, inner setting, individuals, and implementation process) and their individual constructs (Fig 1). The 6 themes are as follows: (1) PCPs’ complex experiences with the centralized LCS program, (2) LCS is o1 priority among many, (3) marked clinician variation in LCS shared decision-making and referral decisions, (4) racial biases and structural inequities, (5) limited clinician knowledge of patient-level screening facilitators, and (6) suggested program improvements. These themes are subsequently elaborated on; representative quotes by theme are shown in Table 2.

**Table 2:** Participant Quotes by Emergent Theme and Subthemes

Major Theme	Subtheme	Participant Quote
PCPs’ complex experiences with the centralized LCS program	Provider knowledge of LCS process	“We place a referral if patients meet the criteria, and the LCS clinic sends them information on how to pursue LCS. . . . I’m not even sure that [city] clinic calls the patients or if they just send them a letter. . . . I’m not sure how much information they get directly from a person other than what I tell them, in [city]. The other thing I understand is that they would have to go to [city] to get the LDCT scan.” (PCP 15)
	Reliance on electronic clinical reminder	“The clinical reminders are pretty complex and they’re based on data that the reminders mine out of CPRS and scheduling package. They don’t always function the way that they’re intended to function.” (LCS staff 1)
	Difficulty connecting with LCS program	“I might get [patients] to have buy-in while they're in my exam room but then when I go ahead and put in a consult, and they’re contacted by somebody they don’t know . . . they can’t really focus on or recall the conversation we had, so their willingness to participate in that study may be less than a discussion in the exam room.” (PCP 13) “Sometimes you forget about it, even though it might be something that you’re interested in, or you're busy and you think you're gonna do it later . . . [they] just don't get around to it.” (LCS staff 1)
	Lack of transparency of LCS program outcomes	“I don't have any sense in terms of efficacy, in terms of like what percent of people are screened or how many lung cancers were prevented to progress to more untreatable variations; that I have no idea.” (PCP 10)
LCS is 1 priority among many	Absence of LCS in HEDIS measures	“Anything that's in the section for the HEDIS reminders probably gets done before I start thinking about things like the lung cancer screening, and that’s just because I know that those are monitored much closer.” (PCP 4)
	Limited time	“Time is always a big one. You’ll get somebody to come in with like 6 things they want to talk about. Almost nobody comes into clinic and goes, ‘Hey, doc, let’s talk about LCS.’” (LCS staff 2)
Marked clinician variation in LCS shared decision-making and referral decisions	Shared decision-making	“We have 120-130 different PCPs here in our system and they all kind of interact along a spectrum. . . . Some of them have a brief conversation with their patients about LCS…Then there are some providers who have a little bit of discussion with their patient about LCS . . . but maybe are a little bit less aggressive . . . more of a like, ‘this is available to you and they’re going to send you some information’ kind of just introducing to the context. . . . I’ve had patients who are very surprised, they call me, they’re scared, they think their doctor thinks they have lung cancer.” (LCS staff 1)
	Influence of patient characteristics	“If you know that . . . they have such bad COPD that you could never do a lung resection on them, then I would question whether doing screening is really useful. . . . If you can’t do anything with the information after they got screened then what would be the point in screening them?” (PCP 14)
	Concern for patient mistrust/lack of perceived benefit/reluctance to additional care	“Some [patients] are so mistrustful they won’t do that [receive treatment or prevention for things not bothering them in the moment]. . . . Some people don’t want to know if they have cancer. . . . Some are too busy to put in the time to get screened. There’s lots and lots of reasons people turn down.” (PCP 7) “Some of it has to do with the patient’s perspective on how much it would benefit them. . . . The people that don’t like having things done and don’t want procedures are probably more likely to say, ‘No, I don’t want it.’” (PCP 4)
Racial biases and structural inequities	Mistrust of health care system among Black veterans	“The only pattern [with medical mistrust] I have seen in general is probably people of color. And it's certainly not most of them, but most of the people that truly will say they don't trust the VA are people of color. . . . I have one patient who sees me about once a year or little less who very clearly says he does not trust the VA. He does not trust me. He quotes the Tuskegee experiments . . . and he is very much convinced that we are here to try and kill off all the Black guys. And he has said as much. Now, like I said, it’s a minority [of patients] but he’s a rather vocal minority.” (PCP 4)
	Culture-specific norms in tobacco products	“Sometimes Black patients smoke something different than cigarettes, like Black & Tans. The guidelines aren’t really clear on that. . . . So if you aren’t smoking cigarettes, they won’t recommend you get the LDCT scan, so maybe that’s why . . . I guess there’s something to be said about systemic issues, or institutional issues, in terms of and how we treat Black and non-Black patients. . . . If you’re not smoking cigarettes, you won’t trigger the reminder. . . .” (PCP 15)
	Provider implicit biases	“Screening disparities come from 2 sources of racism. One would be implicit bias on the part of providers who make assumptions about what Black folk will talk to them about . . . not push as hard to have a shared decision process because they think that’s futile. . . . Despite the fact that every provider believes they’re not racist almost all of us probably are. . . . Institutional socialized racism in the US would naturally create a mistrust of a health care system among people who are on the receiving end of this racism. So, it would not be unexpected for there to be mistrust of the health care system, including mistrust of screening recommendation . . . it’s a vicious cycle.” (PCP 7)
	Systemic barriers exacerbated by race	“My generalization is that our Black veterans are often of a lower socioeconomic class than our White veterans . . . getting that ride to the scanner is just a little bit harder . . . With fewer financial resources, life in general is just a little bit harder and your bandwidth for doing things like preventative health care is gonna be a little bit lower . . . I’d be surprised if it was overt racism . . . My personal experience is that when I talk to Black or White Veterans, the race doesn’t seem to be a big predictor in who is going to be interested in screening or not.” (LCS staff 2)
	Provider lack of knowledge of racial disparities	“I don’t know that there would be any reason why they wouldn’t be referred equally. The VA reminder comes up for anyone that is a smoker or has been a smoker within the past 15 years. It doesn’t matter what color they are, that the reminder is still going to come up, that risk is still going to be there.” (PCP 12)
Limited clinician knowledge of patient-level screening facilitators	Presence of support system	“When they come in to the clinic, the visits with their spouses, they tend to be a little bit more agreeable to the screenings as well because they make a joint decision. . . . Whereas sometimes the person coming on their own will say, ‘Okay, I’ll get it done,’ but then they can’t explain it to their family members once they get home, and so then it causes a lot of confusion. . . . I think when the family member is in . . . it seems to make the veteran feel that they are cared for and also that their health is important, and I think that triggers them to be more proactive in taking care of themselves. . . .” (PCP 1)
Suggested program improvements	Patient-level interventions	“A point-of-care shared decision-making tool would be very helpful. . . . If there was a way to automate that where . . . at the end they could say, ‘Hey, I’m interested in this,’ or, ‘I’m curious but I want more information before I decide.’ . . . For those folks that say, ‘I'm interested in signing up,’ at that point we could just order a scan, and once we order the scan, I think we do pretty well in getting those people into the scanner once that order is placed.” (LCS staff 2)
	Provider-level interventions	“Making sure that the providers know that the rates are higher in the Black population and that’s something that we can mention to our patients . . . [that] might help with getting the providers to refer them more regularly.” (PCP 4) “In [institution], a Black nurse is our RN. Having a diversity of staff creates bridges instead of barriers.” (LCS program staff 3)
	System-level interventions	“When you have a health care disparity you can either do targeted work, you can build some sort of sociocultural intervention that is appropriate and thoughtful and reaches Black veterans, in particular, or you can overcome the global barriers and see if the disparity knocks itself down.” (PCP 7) “If there were more sites available to do it. If you could get your CT scan done at some mobile unit that travels across the state. If there was CT scanning availability in the different CBOCs around . . . if there was same day access for CT scans.” (PCP 2)

CBOC = Community Based Outpatient Clinic; HEDIS = Healthcare Effectiveness Data and Information Set; LCS = lung cancer screening; LDCT = low-dose CT; PCP = primary care provider; RN = registered nurse; VA = veterans affairs.

### PCPs’ Complex Experiences With the Centralized LCS Program

The primary clinician-level contextual factor was how PCPs interacted with the LCS program. The LCS program is a centralized process involving 6 steps: a clinical reminder in the electronic medical record, PCP shared decision-making, PCP referral, LCS coordinator shared decision-making, CT scan scheduling and completion, and CT scan review. PCPs expressed familiarity with the LCS program structure. Most knew details about the LCS process, whereas a few did not know specific details about the process but understood their role to be referring eligible patients. Familiarity with patient engagement with LCS after referral varied from being unsure whether a patient completed LCS to being aware of the reason patients were not participating. Some PCPs followed patients’ CT scan results, whereas others relied on the program to follow up with patients.

PCPs relied on clinical reminders to guide them through eligibility requirements and complete a referral. They determined patient candidacy for referral based on eligibility criteria and life expectancy, comorbidities, and patient priorities. Most PCPs saw the clinical reminder as a facilitator; however, LCS program staff expressed concern about the complexity of the clinical reminders, stating that they do not always function as intended (eg, inaccurate pack-year history).

PCPs and LCS program staff expressed difficulty connecting patients to the LCS coordinator, which had led to veterans being lost to follow-up. They shared concerns that the multistep referral process resulted in potential gaps in care. LCS program staff identified challenges with the program’s adaptation from phoning each veteran referred for LCS to sending mailers to eligible patients to accommodate growing numbers of referrals as a major barrier to LCS uptake. PCPs further emphasized this barrier, describing how patients are often lost to follow-up after being referred to the LCS program due to the onus being on the patient to connect with the screening coordinators.

PCPs were hesitant to outright describe the LCS program as effective, citing consideration of avoidable risks associated with follow-up of LCS (ie, disability resulting from biopsies of benign nodules, psychological impacts of abnormal findings). PCPs also expressed uncertainty about LCS effectiveness because they had not seen program outcomes data; one PCP noted that they had not seen any lung cancer diagnoses identified through the LCS program within their patient panel. However, in terms of ease of use, PCPs’ perceptions were largely positive.

### LCS Is 1 Priority Among Many

PCPs and LCS program staff alike emphasized barriers to introducing LCS during patient visits due to competing priorities. Barriers included time, technology malfunction, screening for other cancers and major health conditions which are included as metrics in the Healthcare Effectiveness Data and Information Set (HEDIS), and LCS eligibility education. Primary among these challenges was limited time during a PCP visit. Time constraints, specifically with shared decision-making, were also evident in PCPs’ interactions with patients.

### Marked Clinician Variation in LCS Shared Decision-Making and Referral Decisions

Many PCPs took pride in the relationships they built with patients and found shared decision-making conversations were easier with patients with whom they had well-established relationships. PCPs sometimes adapted shared decision-making conversations by smoking status when able and appropriate, but did not generally adapt conversations by broad demographic groups (ie, race, age), reiterating that time constraints prevented them from tailoring conversations. LCS program staff acknowledged that PCPs perform shared decision-making differently; some are directive, whereas others have discussions with their patients which vary in style from aggressive to gentle requests.

PCPs used their own discretion to make referrals after assessing their patients’ overall health, with some expressing that certain patients should be excluded from LCS, even if they meet the eligibility criteria. For example, PCPs did not refer patients with multiple or severe comorbidities, those with conditions that dramatically reduce life prognosis (eg, terminal illnesses), nor those that express that they would prefer not to treat any cancer diagnoses. They instead focused on referring patients who were generally in good health, had manageable chronic conditions, had active or prior tobacco use, and/or had a family history of lung cancer.

PCPs also shared factors that made some patients particularly unreceptive to LCS, including mistrust, low perceived risk of lung cancer, and perceived lack of benefit.

### Racial Biases and Structural Inequities

PCPs illuminated several concerns specific to patient race. They cited more prevalent mistrust of the health care system among Black veterans and theorized that culture-specific norms (eg, type of tobacco products used) affected screening rates. They recalled that some Black veterans clarified they used noncigarette tobacco products (eg, cigarillos) which are not factored in the LCS eligibility clinical reminder, exemplifying how technicalities may unintentionally foster disparities in LCS uptake.

PCPs also speculated that implicit bias influenced how other PCPs may interact with patients, positing that bias may manifest as avoiding shared decision-making conversations with Black patients or believing referral to be futile based on beliefs that Black patients would not participate in screening. They also highlighted established systemic and institutional racism as a barrier. A PCP commented that smoking stigma, particularly toward Black veterans, introduces negative perceptions of LCS for some patients, resulting in avoidance or refusal. Other concerns cited as disproportionately affecting Black veterans included socioeconomic barriers (eg, transportation difficulty), physical mobility challenges, missing or unintentionally blocking telephone calls, CT scan costs, perceived importance of LCS, concerns about radiation risks, presence and impact of other health conditions (eg, mental health conditions), and not wanting to receive additional screenings or appointments.

PCPs were not knowledgeable about existing racial disparities in LCS. Most were aware of disparities in other cancer screenings and made inferences that Black patients would also have poorer health outcomes related to lung cancer; however, they clarified that they were unsure.

### Limited Clinician Knowledge of Patient-Level Screening Facilitators

Many PCPs had difficulty identifying facilitators to LCS uptake. Notably, all PCPs who discussed observed facilitators to cancer screening were female, several of whom identified as being of Asian ethnicity. They discussed how having spouses or other family members present at PCP appointments seemed to positively influence interest and participation in LCS.

### Suggested Program Improvements

PCPs and LCS program staff shared several recommendations to address low LCS uptake and disparities in LCS rates. At the patient level, improving the quality and standardization of shared decision-making conversations was a frequently cited opportunity for improvement. Recommendations included introducing a point-of-care shared decision-making tool to help improve standardization and efficiency and facilitating veteran peer interactions with others who have been screened or diagnosed with lung cancer. PCPs also thought that LCS is not well publicized, particularly compared with other cancer screenings. They recommended culturally appropriate and relevant public service announcements, social marketing, or similar interventions specifically targeted toward Black veterans to increase LCS awareness and education.

Recommended clinician-facing interventions included the following: educating providers regarding disparities in LCS uptake and outcomes through continuing education opportunities, sharing hospital- and provider-level LCS statistics so PCPs might better understand how their patient panel is impacted, developing antiracism interventions to address provider implicit bias, and promoting the development and maintenance of patient-provider trust. LCS program staff also identified having a diverse staff as an opportunity to facilitate LCS uptake among Black veterans.

At the system level, PCPs recommended improving access to CT scanners at local sites via mobile units or same-day scans; offering travel assistance or reimbursement; offering patient financial incentives; and improving workflow and capacity by hiring staff and resources specifically tasked with conducting LCS shared decision-making conversations.

## Discussion

To our knowledge, this qualitative study is among the first to describe firsthand clinician perspectives on LCS program implementation and associated racial disparities. The themes identified in this study demonstrate the interdependence between factors (eg, provider knowledge and prioritization, program workflows, patient characteristics) and the pervasiveness of racial biases and structural inequities on the implementation of LCS. It further underscores the need for cross cutting provider-, patient-, and system-level interventions.

At the provider level, PCPs had high levels of awareness and knowledge of LCS recommendations, but reported substantial dependence on electronic clinical reminders and varying quality of shared decision-making given. Multiple studies suggest that knowledge of US Preventive Services Task Force LCS recommendations is not universal among PCPs,^18^^,^^19^ but that clinical reminders can improve LCS uptake.^20^ LCS program staff highlighted how automating portions of shared decision-making may be helpful in not only reducing provider-required time but also in standardizing information sharing. In fact, a variety of such tools have been piloted in different contexts with varying success.^21^, ^22^, ^23^ PCPs also had less knowledge of the LCS process and patient engagement both before and after referral, and thus felt unprepared to help patients navigate scheduling or follow-up difficulties. This highlights the transition from primary care visit to LCS visit as a key area for future interventions.

At the patient level, there was limited knowledge regarding known facilitators for screening (eg, patient knowledge and beliefs about LCS effectiveness, perceived risk, telephone or video clinics for screening, provider endorsement of screening, higher pack-year history).^24^^,^^25^ These facilitators are common to other health interventions (eg, a strong and presumptive physician recommendation leads to better vaccine uptake).^26^ Leveraging these facilitators could improve LCS uptake, especially among patients with higher perceived risk and/or pack-year history.

At the system level, PCPs observed that LCS is not included as a HEDIS metric.^27^ Screening for breast cancer, cervical cancer, and colorectal cancer are all included, however, and PCPs prioritize these and other HEDIS measures over LCS.^28^ Thus, inclusion of LCS in HEDIS will be critical in improving PCPs’ prioritization of LCS shared decision-making and patient uptake, and efforts are currently underway to implement and develop a metric.^29^ Additionally, centralization of the LCS program was a facilitator, with PCPs appreciating the ability to refer and trust that follow-up of results would be handled through the program. Centralized LCS programs are thought to improve annual adherence and disparities among Black populations and other minoritized groups; however, it is unclear which specific components underlie these benefits.^30^^,^^31^ Current guidelines do not recommend one program type over the other, instead advising to choose the type that best matches available resources with local needs. In real-world practice, many programs operate as a hybrid model, incorporating elements of both centralized and decentralized practices (ie, individual providers order CT scans and manage results).^32^ Given this, implementation of a centralized LCS program in health systems in which it is absent may be another important strategy to reduce disparities in LCS.

Importantly, disparities in LCS uptake resulting from racial biases and structural inequities cross cut patient, provider, and system levels. Therefore, interventions focused on these issues will have to also be cross cutting. Although expanded LCS eligibility could help to offset the fact that Black patients develop lung cancers at younger ages and with a lower pack-year history than White patients, PCPs noted that determining accurate pack-year histories can be difficult, especially among veterans who use noncigarette tobacco products.^3^ Furthermore, disparities in screening sensitivities among other minoritized racial and ethnic groups still exist despite expanded eligibility criteria. One potential intervention is to move toward a risk-based screening strategy, which may mitigate racial and ethnic disparities in LCS.^33^^,^^34^ Additionally, implicit biases and structural racism were identified as key factors affecting disparities in LCS. Multiple studies have demonstrated that Black veterans have more negative and discriminatory experiences within the VAHCS and less satisfaction with trust and patient-provider communication.^35^, ^36^, ^37^, ^38^, ^39^ Given that there is limited evidence that interventions to reduce provider implicit biases actually impact outcomes,^40^^,^^41^ efforts should focus on organizational and structural interventions which standardize processes for all patients with close monitoring of how these interventions impact disparities. Additional interventions such as peer support networks may also be an effective strategy.^42^^,^^43^

Strengths of this study include that it directly assessed clinician perspectives on racial disparities in LCS uptake and individual interviews allowed for in-depth discussions and time to generate ideas for improvement. Before this study, our group examined patient perceptions of barriers and facilitators to LCS; these combined insights can now be translated to meaningful interventions to improve LCS uptake and equity in LCS rates. Limitations include that the study was conducted at a single site with a predominantly White- and Black-identifying and male patient population, which may affect its generalizability to other racial groups and to female patients. There was also a lack of racial diversity among clinicians with most self-identifying as White and none self-identifying as Black or Hispanic/Latino. This may have limited ascertainment of facilitators and barriers and contributed to the limited knowledge regarding existing LCS disparities.

## Interpretation

This qualitative study among health care providers at a large VAHCS identified several key barriers and facilitators to LCS uptake, with a specific focus on Black veterans. It demonstrated several promising targets for enhancing equity in LCS (eg, improving the quality of the shared decision-making process through content standardization, better integration of LCS into clinical workflows, streamlining the steps from PCP visit to first CT scan). Future studies should focus on designing and testing such interventions to determine implementation and effectiveness outcomes as they relate to LCS uptake and racial equity in LCS.

## Funding/Support

Research reported in this publication was funded through a Young Investigators Award from the National Comprehensive Cancer Network Foundation.

## Financial/Nonfinancial Disclosures

The authors have reported to *CHEST Pulmonary* the following: N. N. reports funding from the 10.13039/100000060National Institute of Allergy and Infectious Diseases (NIAID), 10.13039/100030717National Comprehensive Cancer Network Foundation, Parker B. Francis Foundation, and LUNGevity Foundation for activities outside the submitted work. I. L. R. received NIH funding outside of the submitted work. L. L. Z. reports current consulting with Eisai and consulting with Novartis within the last 36 months outside the submitted work. None declared (G. K., T. L., A. S., A. B. J., C. E. C., S. S.).

## Supplementary Data

e-Online Data

e-Online Data
