# Supplementary material for: “They Just Don’t Get Around to It”: Clinician Perspectives on Lung Cancer Screening and Racial Disparities Among Veterans
Source: CHEST Pulm. 2025 Mar 18;3(3):100165. doi: 10.1016/j.chpulm.2025.100165 (PMC13418926; doi:10.1016/j.chpulm.2025.100165)
Supplement: e-Online Data [file mmc1.docx]

**Supplement: Sample Interview Questions Mapped to CFIR**

| **CFIR Domain** | **Sample Interview Question** |
| --- | --- |
| **Individual Characteristics** | - What, if anything, do you know about the LCS program that eligible patients can be referred to? - What influences your decision to refer patients to LCS? - Can you please describe your confidence level in discussing risks and benefits of LCS with your patients? |
| **Intervention Characteristics/ Innovation** | - What kinds of changes or alterations do you think would make LCS referrals more effective within your patient panel? - What are some patient characteristics you consider when referring a patient for LCS? - What kinds of changes or alterations do you think would make LCS referrals more effective within your patient panel? |
| **Outer Setting** | - How well do you think the LCS program meets the needs of Veterans? - What barriers do Veterans face when participating in LCS? - What are your patients’ perceptions of LCS? |
| **Inner Setting** | - How important do you think it is to refer patients for LCS compared to the other priorities? - Tell me about resources you use to discuss LCS with patients prior to referral? - How does shared-decision making around LCS work with your practice/patient population? |
| **Race and Racism/  Implementation Process** | - Can you tell me what, if anything, you’re aware of in terms of relative risk for developing lung cancer between Black patients and white patients? - What barriers to screening do you see that exist among Black Veterans? - What facilitators to screening do you see among Black Veterans? - What do you think are some potential ways to increase rates of LCS among the Black Veteran patient population? |
